# Flowerlike Tin Diselenide Hexagonal Nanosheets for High-Performance Lithium-Ion Batteries

**DOI:** 10.3389/fchem.2020.00590

**Published:** 2020-07-29

**Authors:** Qiyao Yu, Bo Wang, Jian Wang, Sisi Hu, Jun Hu, Ying Li

**Affiliations:** ^1^Institute of Advanced Structure Technology, Beijing Institute of Technology, Beijing, China; ^2^School of Materials Science and Engineering, Hebei University of Science and Technology, Shijiazhuang, China

**Keywords:** SnSe_2_, non-stacking, fast ion transfer, nanosheet, lithium-ion battery

## Abstract

SnSe_2_ nanosheet is a common anode for lithium-ion batteries (LIBs), but its severe agglomeration hinders its practical application. Herein, a three-dimensional (3D) SnSe_2_ nanoflower (F-SnSe_2_) composed of non-stacking vertical upward hexagonal nanosheets was prepared through a colloidal method as an anode material for LIBs. Benefiting from the advantages of fast reaction-diffusion kinetics and buffering unavoidable volume variation during cycling, the F-SnSe_2_ electrode displays remarkable specific capacity of 795 mAh g^−1^ after 100 cycles at 100 mA g^−1^ and superior rate performance (282 mAh g^−1^ at 2,000 mA g^−1^). This work provides an effective way to get non-stacking nanosheets in energy storage field.

## Introduction

In recent years, lithium-ion batteries (LIBs) have been widely employed as an energy storage system for portable electronic devices and electric or hybrid vehicles (EVs) due to their high energy density (Jiang et al., 2019; He et al., 2020). However, current LIBs are still hard to satisfy the stringent demand for security, capacity, and cost (Chu et al., 2019; Wang et al., 2019). The anode material is one of the most crucial components that can determine the battery performance directly. Recently, a variety of electrode materials, such as carbon, alloys, and transition metal chalcogenides (TMDs), have been widely investigated as anode materials for LIBs (Chen K. et al., 2018; Zhang et al., 2019; Li et al., 2020). Typically, as one of the most promising anode candidates, Sn-based materials have attracted great attention due to their high theoretical capacity and abundant resource (Lee and Park, 2017). Besides, Se-based and S-based anodes have been widely investigated for LIBs and sodium ion batteries (NIBs) recently due to their high theoretical capacity, fast ion transportation, and decent redox reversibility (Hu et al., 2019; Han et al., 2020). However, there still remains a great challenge owing to its low electronic conductivity and extremely large volume expansion, resulting in poor cyclic stability (Wei et al., 2018).

SnSe_2_ as a typical two-dimensional (2D) layered material has attracted intensive attention as a promising anode material for LIBs owing to its high theoretical capacity, tunable spacing structures, and non-toxicity (Du et al., 2016; Huang et al., 2018). However, inherently poor conductivity and high-cost synthesis methods of SnSe_2_ such as complex reaction routes, high temperature, and some toxic reagents limit practical applications (Chen R. et al., 2018; Ren et al., 2018). One of the most popular approaches is to construct SnSe_2_ nanomaterials with various morphologies and structure, which can effectively improve ion transport kinetics and reduce unavoidable volume expansion. Therefore, it is necessary to search for an effective method to fabricate functional morphology SnSe_2_ materials with a good electrochemical performance.

Herein, we report a facile colloidal synthetic method to prepare three-dimensional (3D) flowerlike SnSe_2_ (F-SnSe_2_). The as-prepared SnSe_2_ is composed of non-stacking vertical upward hexagonal nanosheets, which can restrain self-agglomeration during a chemical reaction, improve electron and ion transport, and accommodate volume expansion during lithium-ion intercalation/extraction. When employed as LIB anode, the F-SnSe_2_ exhibits good lithium-ion storage performance with a high reversible capacity of 795 mAh g^−1^ after 100 cycles at 100 mA g^−1^ and superior rate performances, much better than bulk SnSe_2_ electrode.

## Experimental Section

### Material Synthesis

F-SnSe_2_ was synthesized by a simple colloid method. First, 0.2 mmol of stannous chloride was put into a 25-ml single-neck flash along with 10-ml oleylamine (OAm). The mixtures were magnetically stirred for 30 min at 90°C to form a milky suspension. After the solution was cooled down to room temperature, 0.4 mmol dibenzyldiselenide was added into the mixture and then heated to 240°C for 2 h with a heating rate of 8°C min^−1^. Finally, the obtained black solution was washed with ethanol and cyclohexane several times and vacuum dried at 70°C overnight, and the collected sample was marked as F-SnSe_2_.

### Materials Characterization

X-ray diffraction (XRD) was performed on a Smart-lab using Cu Kα radiation from 10 to 90° at a scan rate of 10° min^−1^. The field-emission scanning electron microscopy (FESEM, Hitachi SU8010) and transmission electron microscopy (TEM, JEOL JEM-2100F) were used to characterize the morphology of the samples. The atomistic structural information and microtopography were characterized using high-resolution (HR) TEM (JEM-2200FS), selected area electron diffraction (SAED), and equipped with an energy-dispersive spectroscopy (EDS) mapping by high-angle annular dark-field scanning transmission electron microscopy (HAADF-STEM). The specific surface area of samples was degassed for 6 h at 200°C, then nitrogen adsorption was done for 20 h by using the Brunauer–Emmett–Teller (BET) (ASAP 2460) method. Raman spectra (Lab RAM-HR Evolution, 532 nm laser) with a power of 0.2 mW and the exposure time of 1 s and X-ray photoelectron spectroscopy (XPS, AXIS ULTRADLD Scientific) were conducted to reveal the chemical compositions and surface electronic states.

### Electrochemical Measurement

The purchased bulk SnSe_2_ and F-SnSe_2_ anodes were prepared by pasting a slurry consisting of active material (70 wt%), acetylene black (20 wt%), and polyvinylidene fluoride (PVDF, 10 wt%) in N-methyl-pyrrolidone (NMP) onto a copper foil current collector. After being dried at 80°C in vacuum overnight, they were cut into disks in the diameter of 10 mm with a mass loading of about 1.1 mg cm^−2^. Then, 2032-type coin cells were assembled in an argon-filled glove box using Whatman GF/D glass fiber filter as a separator, lithium foil as the reference/counter electrode, and electrolyte solution consisted of 1.0 M LiPF_6_ dissolved in 1:1 volume ratio of ethylene carbonate and diethyl carbonate. A LAND CT2001A multichannel battery tester system was used to measure the Li storage performances within the voltage range of 0.01–2.5 V (vs. Li/Li^+^). Cyclic voltammetry (CV, at a scan rate of 0.1 mV s^−1^) tests and the electrochemical impedance spectroscopy (EIS, frequency range of 0.01–100 kHz) were recorded on a CHI 604E electrochemical workstation (Shanghai Chenhua Corp.).

## Results and Discussion

**Figure 1:**
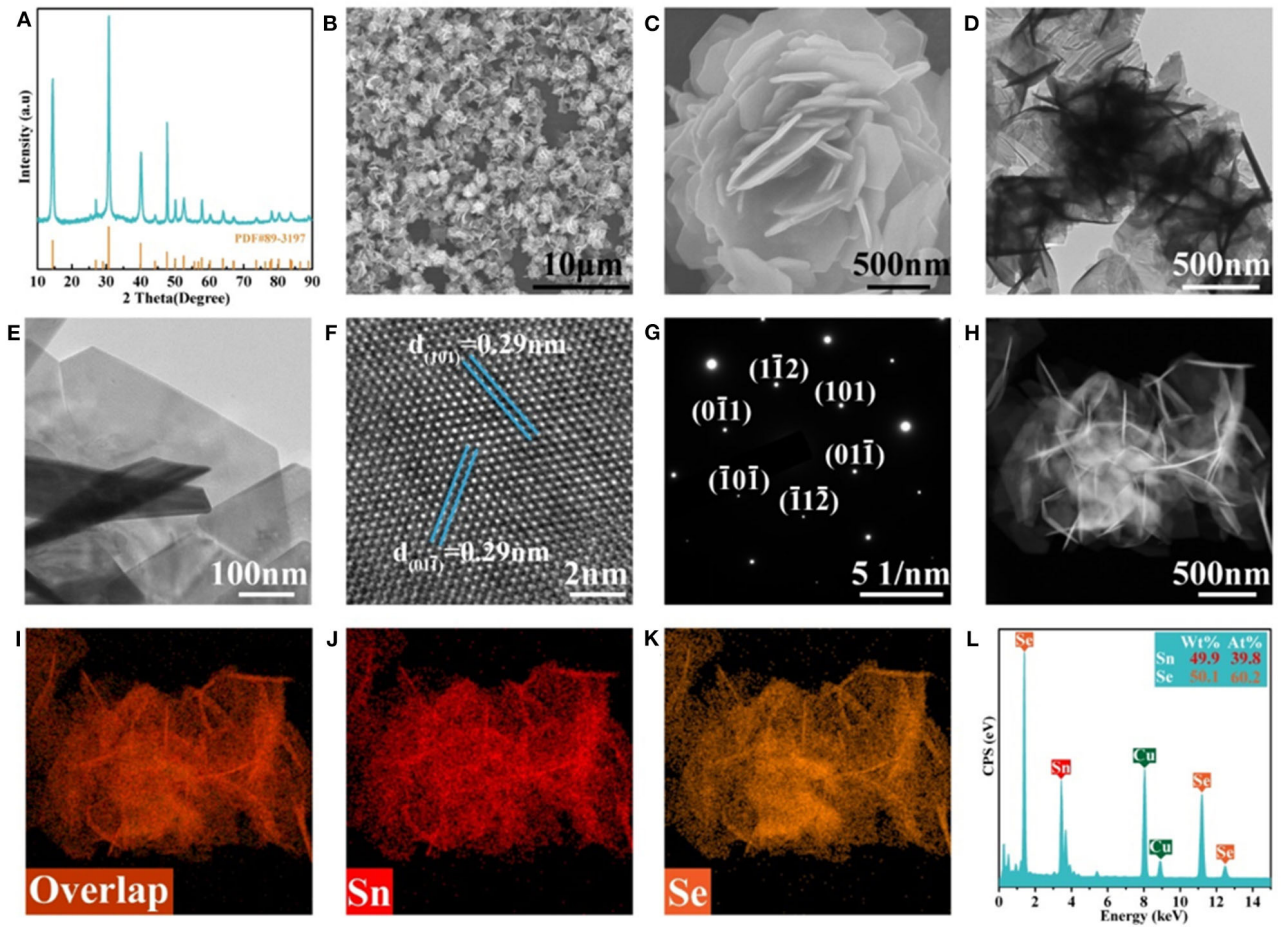
**(A)** X-ray diffraction (XRD) patterns, **(B,C)** field-emission scanning electron microscopy (FESEM) images, and **(D,E)** transmission electron microscopy (TEM) images of F-SnSe_2_. **(F)** High-resolution transmission electron microscopy (HRTEM) image of the F-SnSe_2_ and the corresponding **(G)** selected area electron diffraction (SAED) pattern. **(H)** High-angle annular dark-field scanning transmission electron microscopy (HAADF-STEM) image, **(I–K)** element mapping of Sn and Se and the corresponding **(L)** energy-dispersive spectroscopy (EDS) spectrum.

Figure 1A shows the XRD pattern of the as-prepared F-SnSe_2_ which possesses a rhombohedral crystal structure (JCPDS No. 89-3197) (Gurung et al., 2016), and the sharp diffraction peaks reveal the high-crystallinity SnSe_2_ phase. The morphology and nanostructure of F-SnSe_2_ were investigated *via* the field emission scanning electron microscopy (FESEM) and transmission electron microscopy (TEM). Figure 1B shows the SEM image of SnSe_2_ that presents a uniform nanoflower-like structure, while the purchased bulk SnSe_2_ shows a multilayer stack structure (Figure S1). Figure 1C shows that the F-SnSe_2_ nanoflower shows a self-assembly 3D structure with size in the range of 2–3 μm, which is composed of numerous nanosheets. TEM images (Figures 1D,E) illustrate that the size of F-SnSe_2_ is composed of non-stacking vertical upward hexagonal nanosheets with a size of around 500 nm, which are derived from a preferable growth of SnSe_2_ along (011) crystal plane (Im et al., 2014). Moreover, the detailed microstructure of F-SnSe_2_ is further investigated using high-resolution transmission electron microscopy (HRTEM), as shown in Figure 1F. The (101) and (011) crystal planes with lattice spacing of 0.29 nm (Zhang et al., 2016), which is consistent with the corresponding selected area electron diffraction (SAED) pattern (Figure 1G) that shows well-defined spots, suggest a single crystalline nature of the as-grown F-SnSe_2_. Furthermore, Figures 1H–K display the HAADF-STEM and EDS element mapping of F-SnSe_2_, confirming that Sn and Se elements are distributed uniformly in the whole nanoflower. As expected, EDS analysis (Figure 1L) of F-SnSe_2_ demonstrates that the obtained atomic ratio of Sn to Se approaches 1:2.

**Figure 2:**
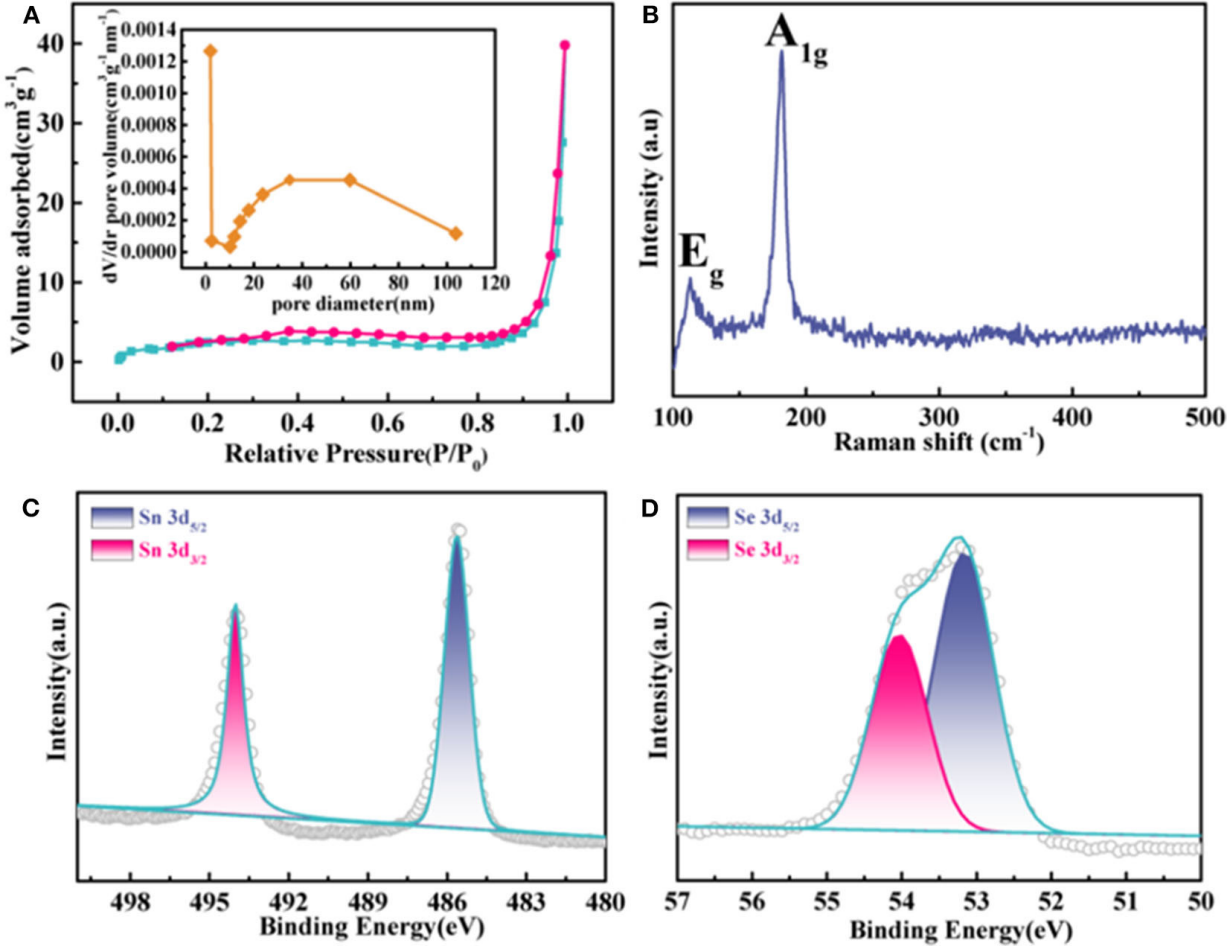
**(A)** N_2_ adsorption–desorption isotherms of F-SnSe_2_; inset is the corresponding pore size distribution curve. **(B)** Raman spectra and high-resolution X-ray photoelectron spectroscopy (XPS) spectra of **(C)** Sn 3d and **(D)** Se 3d in F-SnSe_2_.

Figure 2A exhibits the nitrogen absorption–desorption curve for the F-SnSe_2_ with type IV isotherms, demonstrating the presence of meso/micropore structures. The corresponding Barrett–Joyner–Halenda (BJH) porosity distribution curve is shown Figure 2B, and the calculated specific pore size distribution of F-SnSe_2_ is estimated to be 1–10 nm. Raman spectra (Figure 2B) present two characteristic peaks located at 116 and 185 cm^−1^, corresponding to the Eg and A1g mode of SnSe_2_, respectively (Chen M. et al., 2018). The surface chemical states of F-SnSe_2_ were further studied by XPS as shown in Figure S2. Specifically, in the high-resolution XPS spectrum of Sn 3d (Figure 2C), two peaks located at 485.9 and 494 eV are assigned to 3d 5/2 and 3d 3/2 of typical values of Sn^4+^ ions, respectively (Saha et al., 2016). Correspondingly, two peaks are observed at 53.2 and 54 eV in the Se 3d 5/2 spectrum (Figure 2D), which can be attributed to 3d 5/2 and 3d 3/2 of Se^2−^ in SnSe_2_ (Zhang et al., 2018).

**Figure 3:**
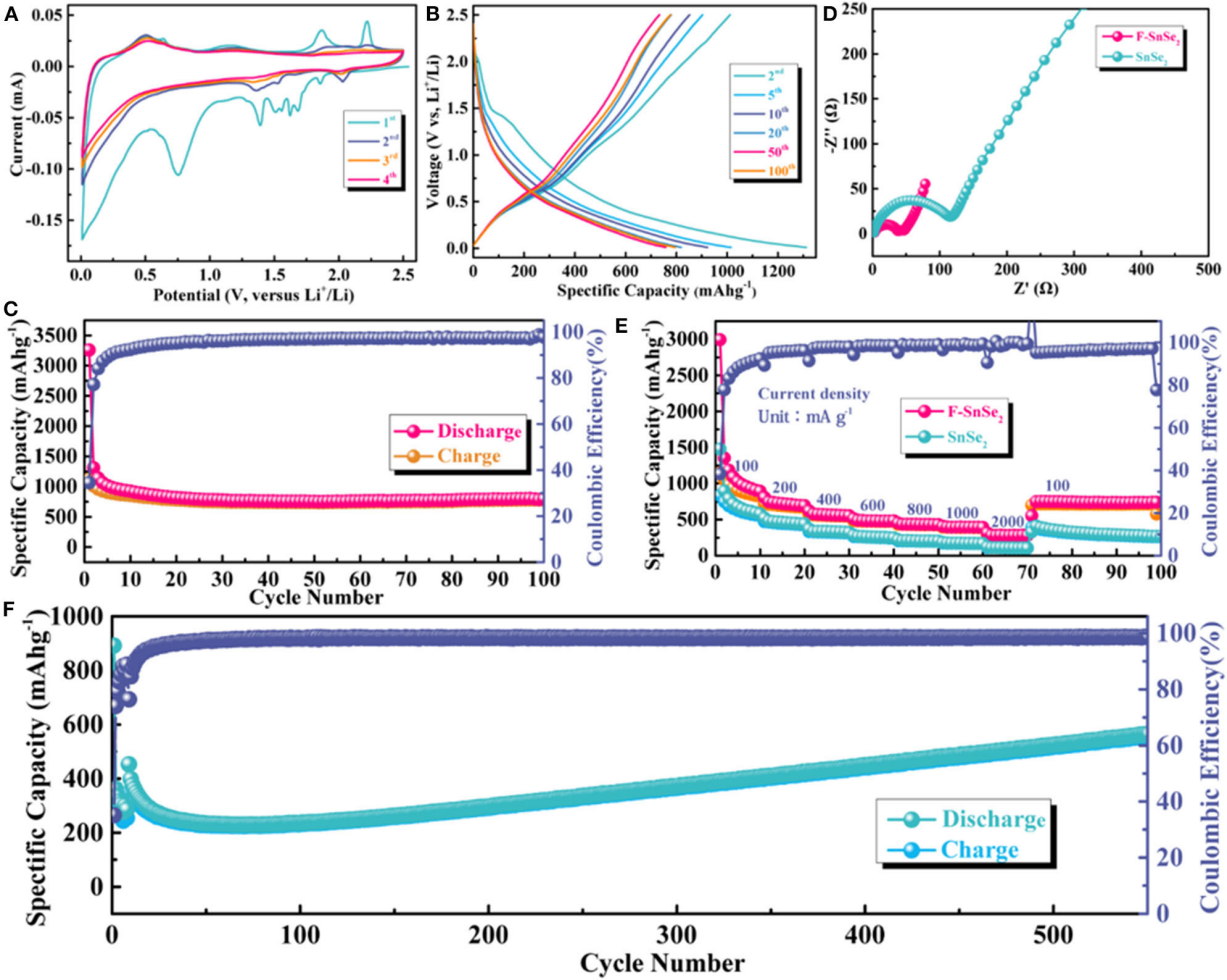
**(A)** Cyclic voltammogram analysis of F-SnSe_2_ between 0.01 and 2.5 V at a scan rate of 0.1 mV s^−1^ for lithium-ion batteries (LIBs). **(B)** Charge and discharge curves from 2nd to 100th cycles of F-SnSe_2_ electrode at a current density of 100 mA g^−1^. **(C)** Cycling performance of F-SnSe_2_ electrode at a current density of 100 mA g^−1^. **(D)** Comparison of electrochemical impedance spectroscopy plots of bulk SnSe_2_ and F-SnSe_2_ electrodes. **(E)** Comparison of rate capability of bulk SnSe_2_ and F-SnSe_2_ electrodes at various current densities from 100 to 2,000 mA g^−1^, respectively. **(F)** Long cycling stability of F-SnSe_2_ electrode at a high current density of 1,000 mA g^−1^.

The lithium storage behaviors of F-SnSe_2_ were evaluated by CV at a scanning rate of 0.1 mV s^−1^ between 0.01 and 2.5 V (Figure 3A). During the first cathodic scan, two large sharp cathodic peaks at around 1.39 and 0.75 V and some small peaks were observed, which disappear in the subsequent cycles, ascribed to lithium intercalation of SnSe_2_ interlayers without phase transition and the formation of irreversible solid electrolyte interphase (SEI) film (Du et al., 2016). The cathodic peaks that shift to 2.03, 1.52, and 1.35 V in the following scans can be assigned to the conversion and alloying reactions (Yuan et al., 2018). Conversely, two pairs of delithiation peaks at around 0.52 and 1.18 V in all cycles are almost completely overlapped, implying good reversibility of the F-SnSe_2_ electrode.

Figure S3 and Figure 3B show the galvanostatic charge–discharge profiles of the F-SnSe_2_ electrode at a current density of 100 mA g^−1^ from the first cycle to 100 cycles. The F-SnSe_2_ electrode delivers an extremely high initial capacity (Figure S3), which is attributed to irreversible side reactions with the electrolyte and the formation of the SEI layer on the surface of the F-SnSe_2_ electrode (Lao et al., 2017). It is worth noting that the initial Coulombic efficiency (CE) should be improved, such as optimizing the electrolyte, pre-lithiation, or compound with carbon matrix to reduce the excess formation of SEI and other irreversible side reactions (Ge et al., 2019; Huang et al., 2020). In addition, the shape of voltage profiles almost overlaps after the initial cycle (Figure 3B), which is consistent with the CV results. As shown in Figure 3C, the F-SnSe_2_ anode exhibits excellent cyclic stability, which delivers a high reversible discharge specific capacity of 795 mAh g^−1^ after 100 cycles at 100 mA g^−1^ with a high CE of nearly 100%, while bulk SnSe_2_ electrode only exhibits a relatively low capacity of 370 mAh g^−1^ after 30 cycles (Figure S4).

Furthermore, EIS was performed with F-SnSe_2_ and bulk SnSe_2_ anode to investigate the kinetic behavior in LIBs. As indicated in Figure 3D, it can be observed that the semicircle of the F-SnSe_2_ electrode is much smaller than that of the bulk SnSe_2_ electrode, which suggests lower charge transfer resistance of the F-SnSe_2_ electrode (Hong et al., 2019). The improved charge transfer is attributed to 3D flower-like nanostructure of F-SnSe_2_ that enhances fast ion transfer kinetics. As expected, the rate performance of F-SnSe_2_ was significantly better than that of bulk SnSe_2_ (Figure 3E). For the F-SnSe_2_ electrode, it could deliver invertible capacity of 892, 694, 554, 483, 430, 393, and 282 mA h g^−1^ at 100, 200, 400, 600, 800, 1,000, and 2,000 mA g^−1^, respectively. When the current density is switched to 100 mA g^−1^ again, a reversible capacity of 745 mA h g^−1^ is recovered. It is necessary to point out that the partial capacity drop in the first 10 cycles for F-SnSe_2_ electrode can be ascribed to stabilization of the SEI film and activation process as well as some irreversible side reactions (Li et al., 2018; Chu et al., 2019). In contrast, the capacities at 100, 200, 400, 600, 800, 1,000, and 2,000 mA g^−1^ are 581, 442, 323, 247, 201, 168, and 106 mA h g^−1^ for bulk SnSe_2_ electrode, respectively. As shown in Figure 3F, the F-SnSe_2_ electrode still delivers a high reversible capacity of 611 mA h g^−1^ after 580 cycles at a high current density of 1,000 mA g^−1^, demonstrating good long-term cycling stability of F-SnSe_2_. It is worth mentioning that the capacity fading in the initial 100 cycles can be ascribed to the repetitive volume expansion/contraction that can fracture the SEI layer and expose new active surfaces for SEI growth (Li et al., 2015). Subsequently, high-rate lithiation-induced mechanical degradation can effectively restructure the 3D SnSe_2_ nanoflower and optimize the SEI, which was defined as reactivation, so the reversible capacity continuously increases during cycling (Qin et al., 2019).

## Conclusion

In summary, 3D flower-like SnSe_2_ has been synthesized *via* a colloidal method. This nanoflower is composed of non-stacking vertical upward hexagonal SnSe_2_ nanosheets, which can improve the ion transfer speed and accommodate the volume expansion during cycling. When evaluated as an anode material for LIBs, the F-SnSe_2_ electrode delivers significantly enhanced electrochemical performance with high capacity and excellent rate performances. A high capacity of 795 mAh g^−1^ at 100 mA g^−1^ after 100 cycles and a remarkable rate capability of up to 282 mA h g^−1^ at 2 A g^−1^ are obtained. Therefore, this unique structure shows a promising prospect to solve the agglomeration problem.

## Data Availability Statement

The raw data supporting the conclusions of this article will be made available by the authors, without undue reservation.

## Author Contributions

BW and QY designed the experiments and wrote manuscript. JW and SH performed all the experiments. JH and YL helped in chemistry experiments and their design, and the chemical analysis. BW, QY, and JW helped in discussion, evaluation, and restructuring of the findings. All authors contributed in manuscript editing.

## Conflict of Interest

The authors declare that the research was conducted in the absence of any commercial or financial relationships that could be construed as a potential conflict of interest.
