# Supplementary material for: Flowerlike Tin Diselenide Hexagonal Nanosheets for High-Performance Lithium-Ion Batteries
Source: Front Chem. 2020 Jul 29;8:590. doi: 10.3389/fchem.2020.00590 (PMC7438772; doi:10.3389/fchem.2020.00590)

**Supplementary Information**

**Flowerlike Tin Diselenide Hexagonal Nanosheets for High-Performance Lithium-ion Batteries**

Qiyao Yu,^a^ Bo Wang^b,^*, Jian Wang^b,^, Sisi Hu^b,^, Jun Hu^a^, Ying Li^a,^*

*^a^ Institute of Advanced Structure Technology, Beijing Institute of Technology, Beijing 100081, China*

*^b^ School of Materials Science and Engineering, Hebei University of Science and Technology, Shijiazhuang 050018, China*

* Corresponding authors Email: wangbo1996@gmail.com (B. Wang), bitliying@bit.edu.cn (Y. Li).

**Figure captions**

Fig. S1. FESEM image of bulk SnSe_2_.

Fig. S2. XPS survey spectrum of the F-SnSe_2_.

Fig. S3. Discharge/charge profiles for the first cycles of F-SnSe_2_ at a current density of 100 mA g^−1^.

Fig. S4 Cycling performance of bulk SnSe_2_ electrode at a current density of 100 mA g^-1^.

**Fig. S1**


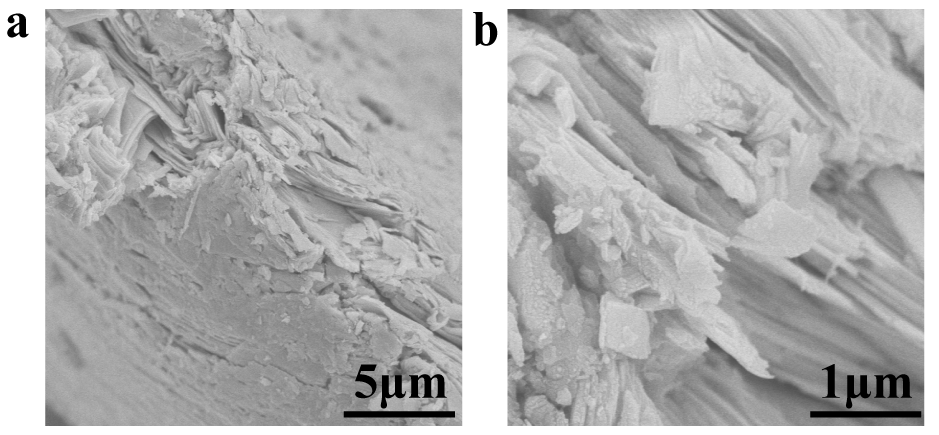


**Fig. S2**


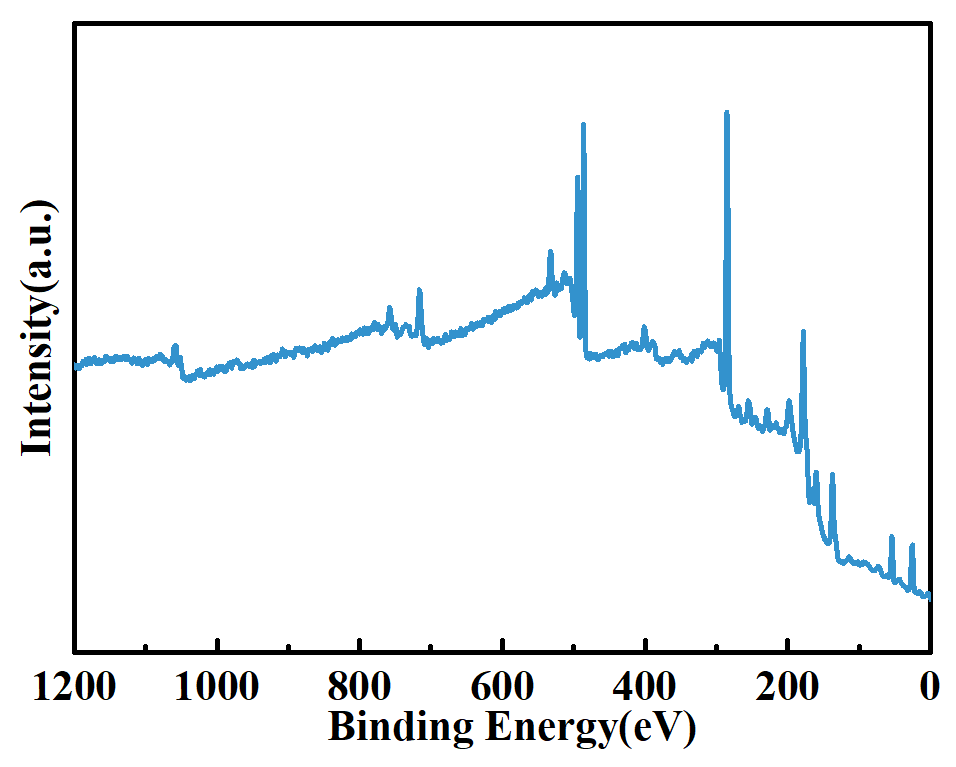


**Fig. S3**


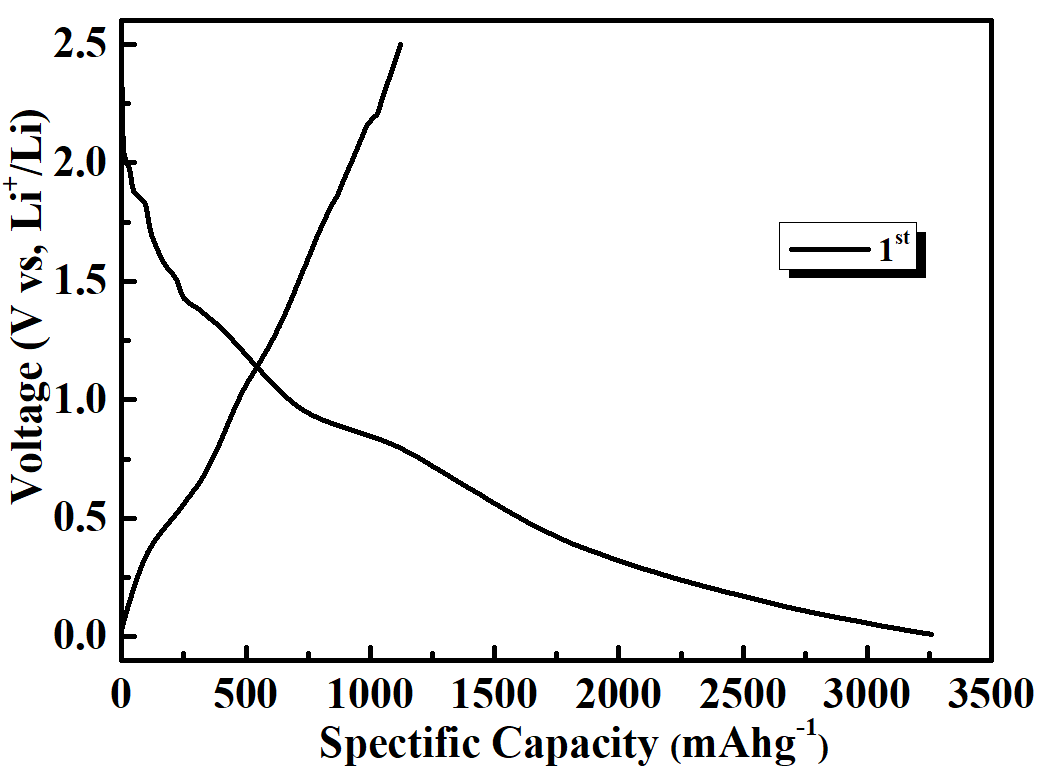


**Fig. S4**


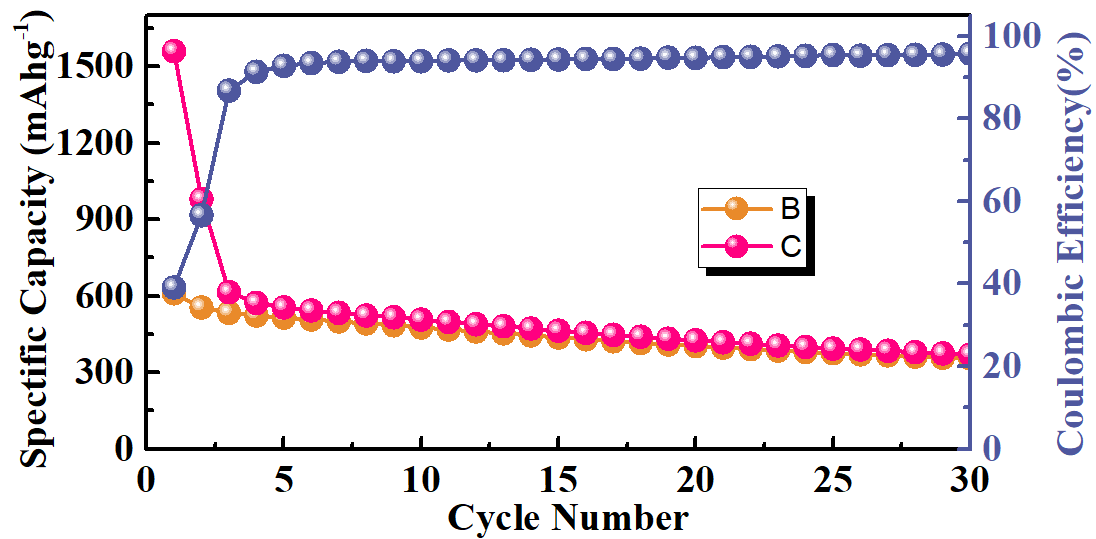

Supplement: Supplementary file 1 [file Data_Sheet_1.docx]
